# Preliminary Real-World Experience with Semaglutide in Obese Patients with Type 2 Diabetes on Chronic Hemodialysis: A Multicenter Pilot Study

**DOI:** 10.3390/medicina62020386

**Published:** 2026-02-16

**Authors:** Alejandra Yugueros, Luis D’Marco, Alejandro Valero, Elena Vivó, Amparo Martínez-Mas, Manuel Calvé, Juan Carlos Alonso, Belén Vizcaíno, Mercedes González-Moya, Ana Checa-Ros, Asunción Sancho, Pablo Molina

**Affiliations:** 1 Servicio de Nefrología Hospital Lluis Alcanyis de Xativa, 46800 Valencia, Spain; aleja2040@hotmail.com (A.Y.);; 2 Grupo de Investigación en Enfermedades Cardiorrenales y Metabólicas, Departamento de Medicina y Cirugía, Facultad de Ciencias de la Salud, Universidad Cardenal Herrera-CEU, CEU Universities, 46113 Valencia, Spain; luis.dmarcogascon@uchceu.es (L.D.); ana.checaros@uchceu.es (A.C.-R.); 3 Servicio de Nefrología, Hospital Doctor Peset, 46017 Valencia, Spainsanch_asucal@gva.es (A.S.); 4 Servicio de Nefrología, Hospital de la Ribera, 46600 Alzira, Spain; luisgerardodg@gmail.com; 5 Servicio de Nefrología, Hospital Universitari i Politècnic La Fe, 46026 Valencia, Spain; 6 Departament de Medicina, Facultat de Medicina i Odontlogia, Universitat de Valencia, 46010 Valencia, Spain

**Keywords:** chronic kidney disease, hemodialysis, diabetes, obesity, GLP1-RA, semaglutide

## Abstract

*Background and Objectives:* Semaglutide is a glucagon-like peptide-1 receptor agonist (GLP-1RA) that has demonstrated metabolic and weight benefits in diabetic and obese patients with chronic kidney disease (CKD) who are not on dialysis; however, evidence in the hemodialysis (HD) population is scarce. Weight control and body composition optimization are particularly challenging in HD because of fluid shifts and the risk of protein-energy wasting. *Materials and Methods:* This prospective, multicenter, real-world, uncontrolled observational pilot study explored the short-term safety and changes in anthropometric and body-composition parameters after semaglutide initiation in obese adults with type 2 diabetes mellitus (T2DM) undergoing chronic HD. Patients were assessed at baseline and at 3 and 6 months. The primary endpoint was the change in body mass index (BMI), dry weight, and fat mass assessed by bioimpedance spectroscopy (BIS). *Results:* Thirteen patients were included (10 male, 77%), with a median age of 61.9 years (IQR 55–69). Semaglutide was started at 0.25 mg/week and titrated up to 1 mg/week according to tolerance. Three patients (23.1%) experienced transient nausea that was resolved over time or after dose adjustment, without discontinuation. From baseline to month +6, BMI decreased by a median of 1.5 kg/m^2^ and dry weight by 5.0 kg, mainly driven by a median reduction in fat mass of 9 kg; lean tissue mass and serum albumin did not change significantly. *Conclusions:* In this small, uncontrolled exploratory study, semaglutide was generally well tolerated and was associated with short-term reductions in body weight and fat mass in obese patients with T2DM on HD. These findings are hypothesis-generating and require confirmation in larger controlled prospective studies to define safety and clinical benefit in this population.

## 1. Introduction

Diabetic nephropathy is the leading cause of end-stage renal disease (ESRD) requiring renal replacement therapy (RRT). Around 40% of the population with type 2 diabetes mellitus (T2DM) will develop chronic kidney disease (CKD) during their illness and from then, a significant number of patients will progress to RRT need [1].

The mortality in patients with diabetes on hemodialysis (HD) has increased, mainly due to cardiovascular and infectious causes [2] associated with other morbidity risk factors, such as arterial hypertension, smoking, obesity, and uremic-related factors, like high interdialytic weight gain, which is also a predictor of mortality in this population [3]. Among these factors, weight control is a critical feature in the daily prescription of HD sessions, since both excessive ultrafiltration and fluid overload increase the mortality risk [4]. Thus, beyond fluid overload, body composition abnormalities—particularly excess adiposity and sarcopenic obesity—are increasingly recognized as relevant contributors to adverse outcomes in patients undergoing HD. Obesity in this population is a complex and heterogeneous condition, often characterized by an increase in fat mass with a relative or absolute reduction in lean mass, which may negatively influence metabolic control, inflammation, nutritional status, and cardiovascular risk. Therefore, strategies aimed at improving weight and body composition, rather than focusing solely on absolute body weight, are of growing clinical interest in dialysis populations.

Regarding the treatment of T2DM patients with CKD, there are different consensus guidelines, such as the KDIGO 2020 Clinical Practice Guideline for Diabetes Management in CKD [2]; however, there are few recommendations regarding the treatment of T2DM in HD patients, due to the scarce evidence in this population [5,6]. Currently, most of the patients switch to insulin once they initiate dialysis. It is knowing the lipogenic effects of this hormone are associated with the consequent hypoglycemic risk of these therapies. Moreover, insulin therapy in patients on HD is frequently associated with weight gain, increased fat mass, and glycemic variability, partly related to reduced renal insulin clearance and fluctuations in insulin sensitivity during and between dialysis sessions. These factors complicate glycemic management and may further exacerbate obesity-related metabolic disturbances, reinforcing the need for alternative glucose-lowering strategies that provide metabolic benefits beyond glycemic control.

Semaglutide is a glucagon-like peptide-1 receptor agonist (GLP1-RA) hypoglycemic medication that has shown excellent results in diabetic and obese patients with CKD who are not under dialysis treatment [7]. Nonetheless, in the HD population, there is little evidence of the efficacy of these types of drugs; furthermore, there is special concern about their pharmacokinetics and possible gastrointestinal adverse effects.

GLP1-RAs have demonstrated pleiotropic effects, including weight reduction, appetite regulation, improvement in insulin sensitivity, and potential cardiovascular and anti-inflammatory benefits. These properties are particularly attractive in the HD population, where cardiovascular disease remains the leading cause of mortality, and therapeutic options targeting both metabolic and cardiovascular risk factors are limited. However, the exclusion of dialysis patients from most randomized clinical trials has resulted in a significant evidence gap regarding the efficacy, safety, and tolerability of GLP1-RAs in this setting.

Hence, this study aimed to analyze the effect of the GLP1-RA semaglutide on body mass index (BMI), dry weight, fat mass, and other metabolic and nutritional markers, as well as the safety of this medication in a population of T2DM and obese patients on HD. By addressing these aspects, our study seeks to provide real-world evidence on the potential role of semaglutide as a therapeutic option for metabolic management in patients with T2DM undergoing HD, an underrepresented and high-risk population for whom optimized treatment strategies are urgently needed.

## 2. Materials and Methods

### 2.1. Study Design

This is a prospective real-world multicenter study in diabetics and obese patients on HD, who started treatment with semaglutide for metabolic and weight control from May 2023 to April 2024. The recruitment centers were the Hospital Universitario Dr. Peset (Valencia), Hospital Lluís Alcanyis (Xátiva), Hospital de la Ribera (Alzira), and CEU Cardenal Herrera University in Valencia, Spain.

### 2.2. Inclusion and Exclusion Criteria

All participants were ≥18 years old and had T2DM and obesity, defined as BMI > 30 kg/m^2^ at the time of semaglutide initiation. Exclusion criteria included refusal to provide informed consent and active inflammatory conditions such as infection, active cancer, or other inflammatory states.

Eventually, thirteen patients consented to participate after being informed about the benefits and risks associated with the study. The antidiabetic medication was adjusted according to the technical data sheet before treatment onset: inhibitors of dipeptidyl peptidase 4 (i-DPP4) were suspended, and insulin dosage was reduced.

The research was conducted following the Declaration of Helsinki as revised in 2013. The Research Ethics Committee approved the study from the participating hospitals. Authorization was gathered from the Biomedical Research Ethics Committee at CEU Cardenal Herrera University (approval code: CEEI23/424; approval date: 23 May 2023). Informed consent was obtained from all patients before being included in the study.

### 2.3. Semaglutide Treatment Protocol

Treatment with subcutaneous semaglutide administered weekly was initiated with 0.25 mg to assess tolerance and potential adverse effects for 4 weeks. If tolerated, the dose was increased to 0.5 mg for 4 additional weeks and then to a target dose of 1 mg/week. Dose reductions or slower titration were allowed in case of gastrointestinal intolerance and were managed clinically; follow-up assessments were performed regardless of the achieved dose.

### 2.4. Study Variables

A clinical assessment, analytical test, and a baseline bioimpedance analysis were performed at 3 and 6 months of the follow-up period. Primary endpoints included changes from baseline to the end of the study in BMI, dry weight, and fat mass as measured by bioimpedance. Secondary endpoints were changes from baseline in other parameters, such as the lean tissue mass (LTM), total body water (TBW), extracellular water (ECW), overhydration (OH), and serum albumin levels. Moreover, other analyzed markers included serum levels of hemoglobin, total protein, glucose, and glycosylated hemoglobin.

Data related to the HD technique were also collected, including interdialytic weight gain and the estimated dialysis dose using the second-generation Kt/V Daurgidas score, following the formulas previously described [8,9].

Body composition and hydration status were assessed with whole-body spectroscopic bioimpedance measurements (BCM; Fresenius Medical Care, Bad Homburg, Germany) performed before a mid- or late-week HD session, following manufacturer recommendations. To improve comparability, measurements were scheduled at similar points relative to dialysis at baseline and follow-up visits and were performed by trained staff at each center. Laboratory samples were drawn before the mid-week session.

### 2.5. Statistical Analysis

Descriptive analyses summarize patient characteristics. Quantitative variables are presented as means (SD) or medians with interquartile ranges (IQR), depending on distribution. Qualitative variables are presented as counts and percentages. Changes over time were explored with paired non-parametric Wilcoxon signed-rank tests; *p*-values are reported without adjustment for multiple comparisons because this is an exploratory pilot study. No missing data occurred for the variables reported at baseline, 3 months, and 6 months.

Statistical analysis was performed using IBM SPSS Statistics v20 (IBM Corporation, New York, NY, USA).

## 3. Results

### 3.1. Characteristics of the Patients

The study included 13 patients, ten of whom were females (77%). The median age was 61.9 years (IQR 55–69). Baseline characteristics are presented in Table 1. The most prevalent CKD etiology was diabetic kidney disease (DKD) (n = 9; 69%). Among T2DM complications, nine patients (69%) presented retinopathy, five (38.5%) neuropathy, and four (30.8%) vasculopathy (peripheral vascular disease).

Most patients presented several cardiovascular risk factors or established cardiovascular disease: heart failure in seven of them (53.8%), of which three (23%) patients presented chronic ischemic heart disease; four (30.8%) suffered from cerebrovascular disease and all patients (100%) presented dyslipidemia.

Regarding antidiabetic treatments at the onset of recruitment: ten patients (76.9%) were receiving insulin, which was reduced in all patients during the 6 months of follow-up, and six (46.2%) of them were receiving meglitinide analogs. iDPP4 medication was withdrawn according to the datasheet at baseline and six (46.2%) patients were receiving meglitinides that were maintained during follow-up.

### 3.2. Semaglutide Efficacy

**Figure 1:**
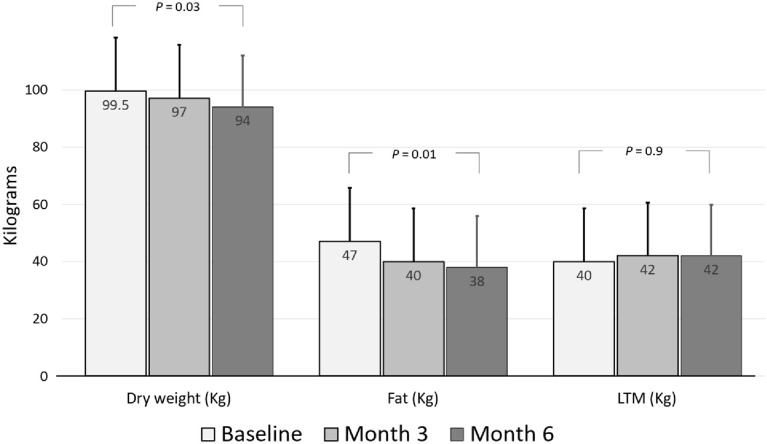
Changes in dry weight, fat mass, and lean tissue mass (LTM) throughout semaglutide treatment. Central values at each time point correspond to the cohort summary shown in Table 2 (medians with IQR). Dispersion measures are reported in Table 2.

The baseline characteristics of the patients, at 3 months and 6 months of the follow-up period, are presented in Table 2. After 6 months of treatment with semaglutide, there was a significant decrease in weight and BMI of 5 kg and 1.5 kg/m^2^, respectively (Figure 1).

Regarding secondary outcomes, weight loss appeared mainly at the expense of fat mass, whereas lean tissue mass did not change significantly over follow-up (Figure 1 and Table 2). This pattern was consistent across individual patients, with no evidence of clinically relevant loss of fat-free mass at any time point. At the metabolic level, fasting glucose, glycated hemoglobin, and inter-dialytic weight gain decreased (Table 2).

These metabolic improvements were observed early after treatment initiation and were maintained throughout the follow-up period. No relevant changes were observed in other nutritional parameters. Blood pressure, serum hemoglobin, and Kt/V remained unchanged during the follow-up period. Dialysis prescription and adequacy parameters were stable, and no treatment modifications were required during the study period.

Concerning tolerability and adverse effects, three (23.1%) of the patients presented transient nausea that subsided with time or dose adjustment, without forcing treatment suspension. No patients required dose discontinuation due to intolerance, and treatment adherence was maintained throughout the entire 6-month follow-up period. No major adverse effects (death or hospitalization) attributed to the use of semaglutide were observed, nor were there any episodes of pancreatitis. Additionally, no episodes of hypoglycemia or clinically relevant changes in laboratory safety parameters were recorded.

## 4. Discussion

In this small, uncontrolled cohort, semaglutide initiation was associated with a reduction in body weight predominantly driven by decreased fat mass, while lean tissue mass and other nutritional parameters remained broadly stable over 6 months. Tolerability was acceptable, with only minor gastrointestinal adverse events reported. Given the exploratory design and absence of a control group, these observations should be interpreted cautiously and cannot establish causality.

Nevertheless, the consistency of the observed changes across multiple anthropometric and body composition parameters suggests a biologically plausible effect of semaglutide in this population. Importantly, the preservation of lean tissue mass alongside fat mass reduction is a clinically relevant finding in patients on HD, in whom protein-energy wasting and sarcopenia are strongly associated with adverse outcomes. From a clinical perspective, interventions capable of improving adiposity without compromising nutritional status may represent a meaningful advance in the management of obese patients undergoing HD.

Since their introduction, GLP1-RA drugs have shown excellent results in T2DM and more recently in CKD patients not on dialysis (FLOW study), with cardiovascular safety studies supporting them in these populations [10,11,12]. In addition, these drugs induce significant weight loss without producing episodes of hypoglycemia, which constitutes an additional advantage for patients. GLP1-RAs can be classified into two groups: incretin mimetics and analogs of human GLP-1. The former is eliminated mainly by the kidneys; therefore, its excretion is reduced in CKD patients. The latter are eliminated via peptidases in different tissues, so their elimination does not depend on renal function, making them appropriate to be used in patients on HD [13,14].

These pharmacological characteristics are particularly relevant in patients receiving HD, in whom altered drug clearance, polypharmacy, and metabolic instability complicate therapeutic decision-making. The renal-independent elimination of human GLP-1 analogues supports their theoretical suitability for use in advanced CKD and HD, provided that safety and tolerability are adequately demonstrated.

However, the glomerular filtration rate threshold for GLP1-RA treatment so far is 15 mL/min/1.73 m^2^, due to a lack of evidence in TRR patients. However, some series reported a few cases of patients in HD under GLP1-RA treatment. Marbury et al. [14] studied semaglutide in diabetic patients with CKD at different stages (mild, moderate, severe, and RRT), postulating that HD does not affect pharmacokinetics, nor did they observe changes in safety parameters or significant adverse effects. The authors concluded that semaglutide could be a useful treatment for CKD patients regardless of their renal function, including those on HD [14]. In a similar investigation, Osonoi et al. [15] evaluated the safety and efficacy of liraglutide in diabetic patients at different stages of CKD (including HD patients). The authors observed the absence of changes in pharmacokinetic parameters in those patients on HD. The drug was well tolerated, without any evidence of relevant adverse effects. Therefore, they concluded that liraglutide could be used safely in HD patients [15,16,17].

Our findings are consistent with these previous observations and extend them by providing additional information on body composition and nutritional parameters over a medium-term follow-up. While previous reports primarily focused on pharmacokinetics and safety, our study contributes complementary clinical data suggesting that semaglutide may exert favorable metabolic effects in HD patients without compromising nutritional status.

In our study, we observed that tolerability was adequate throughout a 6-month follow-up. Only 3/13 patients had minor adverse effects (nausea and/or vomiting) without requiring hospitalization or mortality associated with the use of the drug in any case, and no episodes of hypoglycemia were reported. Therefore, our results are in line with previous investigations [15,16,17].

The absence of hypoglycemic episodes is particularly noteworthy in this population, given the high baseline risk of hypoglycemia in patients on HD due to impaired insulin clearance and fluctuating insulin sensitivity. This safety profile may represent a relevant advantage over insulin-based strategies, especially in patients with complex glycemic patterns.

According to our results, a significant weight loss (−5 kg) was observed after the initial 6 months of the follow-up period; mainly at the expense of fat mass (no reduction in lean mass was observed). In this regard, a paradoxical phenomenon of obesity in HD has been supported; in this phenomenon and contrary to the general population, those patients on dialysis with higher BMI have higher survival rates. Nevertheless, this association is not entirely clear, and it is known that muscle mass appears to be greater than fat to confer this improvement in terms of survival [18]. On the contrary, an increase in organ-specific fat deposits, such as the epicardial adipose tissue, has been reported as an independent risk factor for coronary artery disease in HD patients [19]. Of interest, weight loss at the expense of fat mass should represent a greater opportunity for HD patients to shorten the time to enter the transplant waiting list, especially in patients with severe obesity [20,21].

In this context, our findings support the concept that not all weight loss is detrimental in HD patients and that selective reduction in fat mass, particularly ectopic or organ-specific adiposity, may be beneficial. From a practical standpoint, achieving clinically meaningful fat mass reduction while preserving lean tissue may help reconcile the so-called “obesity paradox” with the need to optimize cardiovascular risk and transplant eligibility in this population.

The reduction in basal glycaemia and glycosylated hemoglobin entails a decrease in the interdialytic weight gain, implying this weight improvement and better tolerance to HD sessions. Kondo et al. [22,23] already described this effect, in a cohort of HD patients after a 3-month follow-up. Moreover, a reduction in insulin doses, and in some cases, the withdrawal, implies multiple beneficial effects for HD patients, such as reducing the risk of hypoglycemia, reducing weight gain, sodium, and water reabsorption while maintaining adequate glycemic control [24]. All these advantages make us assume that this group of drugs (GLP1-RA) could be safe and useful in HD patients, suggesting that they could serve as a complementary therapy to the current management of these patients [25]. Taking together, these metabolic and clinical effects suggest that GLP1-RAs may contribute not only to glycemic control but also to improved volume management and dialysis tolerance, which are key determinants of quality of life and outcomes in HD patients. As such, their role may extend beyond glucose-lowering, positioning them as potentially valuable adjuncts in the multidisciplinary management of obese patients with T2DM on HD.

Our study had several limitations. The most important are the study sample and the lack of determination of more serum metabolic dysfunction and/or inflammatory markers (insulin, c-peptide, adipokines, interleukins, etc.). Moreover, we did not control dietary intake changes and/or other nutritional parameters. Despite these limitations, the present study provides real-world evidence in a population largely excluded from randomized clinical trials. These preliminary findings may serve as a basis for future controlled studies aimed at confirming the metabolic and nutritional effects of semaglutide in HD patients and defining its optimal role within current treatment algorithms.

## 5. Conclusions

In conclusion, this prospective exploratory real-world pilot study suggests that semaglutide may be feasible and generally well tolerated for short-term weight management in obese patients with T2DM undergoing chronic hemodialysis, with observed weight reductions largely attributable to decreased fat mass. Because of the small sample size, short follow-up, and lack of a control group, these findings are hypothesis-generating. Larger controlled prospective studies are needed to confirm long-term safety, effects on body composition (including lean mass), and clinical outcomes in the hemodialysis population.

## Figures and Tables

**Table 1:** Patients baseline characteristics.

Characteristic	N = 13
Age (median years)	61.9 (IQR 55–69)
Gender Female/Male, n (%)	10/3 (77/23)
**Etiology CKD, n (%)**	
Diabetic nephropathy	9 (69)
Others	4 (31)
**T2DM Complications, n (%)**	
Diabetic neuropathy	5 (39)
Diabetic retinopathy	9 (69)
Diabetic vasculopathy	4 (31)
**Comorbidities, n (%)**	
Hypertension (HTA)	11 (84)
Dyslipidemia	13 (100)
Heart Failure	7 (54)
Ischemic Heart Disease	3 (23)
**Previous Antidiabetic Treatments, n (%)**	
Insulin	10 (76)
DPP-4 inhibitors (iDPP4)	6 (46)
Meglitinide analogs	6 (46)

**Table 2:** Patients’ parameters at baseline and during the follow-up.

Parameter	Baseline (n = 13)	Month +3 (n = 13)	Month +6 (n = 13)	*p*-Value
BMI (kg/m^2^)	34.5 (31–39) *	34 (31–37)	33 (29–37)	0.04
Dry weight (kg)	99.5 (92–106)	97 (92–104)	94 (89.5–101)	0.03
Systolic Blood Pressure (mmHg)	145 (132–160)	144 (128–161)	149 (138–157)	0.58
Diastolic Blood Pressure (mmHg)	77 (64–80)	76 (61–81)	75 (66–81)	0.81
Interdialytic Weight Gain (kg)	3 (1.6–3.7)	2 (1.7–2.8)	2 (1.5–2.7)	0.03
LTM-Bioimpedance (kg)	40 (25–49)	42 (26–60)	42 (32–63)	0.91
OH-Bioimpedance (kg)	1 (0.4–1.4)	0.6 (0.6–1.7)	1.3 (0.5–2.1)	0.7
FAT-Bioimpedance (kg)	47 (35–57)	40 (32–51)	38 (32–52)	0.01
Standard Kt/V	1.9 (1.3–2.2)	1.8 (1.3–2)	1.6 (1.2–2.0)	0.77
Albumin (g/dL)	3.9 (3.6–4.3)	3.8 (3.4–4.2)	3.8 (3.3–4.1)	0.3
Proteins (g/dL)	6.6 (6.3–7)	6.6 (6.2–7)	6.5 (6.4–6.8)	0.29
Glycated Hemoglobin (%)	6.6 (5.3–8)	6.1 (5.2–6.6)	6.2 (6.2–6.7)	0.49
Fasting Glucose (mg/dL)	163 (115–187)	125 (94–153)	131 (104–164)	0.36
Hemoglobin (g/dL)	11.9 (10.7–13)	11.4 (10.4–12.4)	11.8 (11.1–12.4)	0.49
Hematocrit (%)	36 (32–40)	37 (31–38)	36 (34–38)	0.84

BMI, body mass index; LTM, lean tissue mass; OH, overhydration; Kt/V, dialysis adequacy. *p*-values correspond to paired comparisons of baseline versus month +6 using Wilcoxon signed-rank tests (exploratory, no multiple-comparison adjustment). * continuous variables are reported as median (IQR).

## Data Availability

The data presented in this study are available on request from the corresponding author due to ethical and privacy restrictions associated with the prospective observational design and the use of patient clinical data.
